# Chitosan/hyaluronic acid/plasmid-DNA nanoparticles encoding interleukin-1 receptor antagonist attenuate inflammation in synoviocytes induced by interleukin-1 beta

**DOI:** 10.1007/s10856-018-6160-3

**Published:** 2018-10-01

**Authors:** Rong-Hui Deng, Bo Qiu, Pang-Hu Zhou

**Affiliations:** Department of Orthopedics, Renmin Hospital of Wuhan University, Ziyang Road 99, Wuhan, 430060 China

## Abstract

Synovial inflammation mainly resulting from interleukin-1 beta (IL-1β) plays a crucial role in the early and late stage of osteoarthritis. Recent progress in therapeutic gene delivery systems has led to promising strategies for local sustained target gene expression. The aim of this study was to design a nanoparticle made of chitosan (CS)/hyaluronic acid (HA)/plasmid-DNA (pDNA) encoding IL-1 receptor antagonist gene (pIL-1Ra) and furtherly use it to transfect the primary synoviocytes, and then investigate whether CS/HA/pIL-1Ra nanoparticles could make the synoviocytes overexpress functional IL-1Ra to attenuate inflammation induced by IL-1β. In this study, CS was modified with HA to generate CS/HA nanoparticles and then combined with pIL-1Ra to form CS/HA/pIL-1Ra nanoparticles. The physicochemical characteristics results showed that CS/HA nanoparticles exhibited an appropriate particle size (144.9 ± 2.8 nm) and positive zeta potential ( + 28 mV). The gel retardation assay revealed that pDNA was effectively protected and released in a sustained manner more than 15 days. Cytotoxicity results showed that CS/HA/pIL-1Ra nanoparticles had a safe range (0-80 μg/ml) for the application to synoviocytes. RT-qPCR and western blot analysis demonstrated that CS/HA/pIL-1Ra nanoparticles were able to increase IL-1Ra expression in primary synoviocytes, and reduce the mRNA and protein levels of matrix metalloproteinase-3 (MMP-3), matrix metalloproteinase-13 (MMP-13), cyclooxygenase-2 (COX-2) and inducible nitric oxide synthase (iNOS) in IL-1β-induced synoviocytes. Our findings indicated that CS/HA/pIL-1Ra nanoparticles efficiently transfected synoviocytes and attenuated synovitis induced by IL-1β, which will provide a potential strategy for OA synovitis.

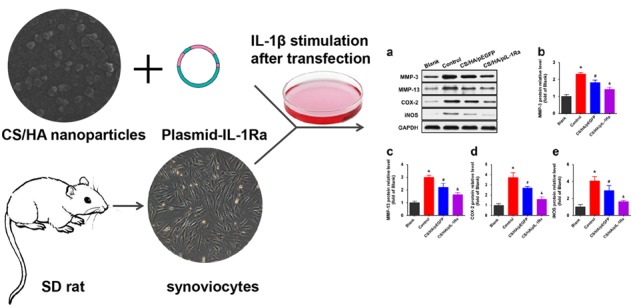

## Introduction

Osteoarthritis (OA), a main cause of disability in elderly people, is a very common disease of joints that represents an enormous burden on individuals and society [1, 2], and now considered involving the whole joint, such as the degeneration of cartilage, subchondral bone remodeling and synovial inflammation [3]. Importantly, synovial inflammation plays a crucial role in the early and late stage of OA which leads to the production and release of inflammatory cytokines and mediators, resulting in the destruction of homeostasis for accelerating matrix degeneration and cell death [4]. Interleukin-1 beta (IL-1β) is regarded as one of the main cytokines involved in the pathogenesis of synovial inflammation [5]. It mediates cell signaling by binding the membrane receptors named IL-1R type I of synoviocytes and chondrocytes, leading to the production of other cytokines, adhesion molecules, enzymes, and inflammatory mediators [6]. Consequently, blocking this signal pathway in synoviocytes presents a promising therapeutic strategy to prevent synovial inflammation in OA [7].

Cytokine IL-1 receptor antagonist (IL-1Ra), a competitive inhibitor of IL-1β without triggering an agonist response, has been shown as a therapy to treat OA through local intra-articular injections [8, 9]. But, the intra-articular injection of therapeutic drugs has a poor anti-inflammatory effect because of the rapid clearance [10]. Gene therapy is a successful technology to overcome this problem, as it offers the advantage of inducing local sustained expression, and a number of proof-of-principle experiments have also been carried out with retroviral and adenoviral vectors containing potential anti-arthritic genes [11]. Viral vectors have high transfection efficiency but they still have many adverse effects such as immune responses and unexpected gene mutations [11, 12]. Meanwhile, non-viral vectors are being specifically developed to serve as safer alternatives, due to its high security, simple synthesis and methods for mass production [13].

Among non-viral vectors, chitosan (CS), a natural cationic polysaccharide which allows it to complex DNA molecules, is considered to be an ideal carrier material because of its low immunogenicity, non-toxicity, and biodegradability [14]. Nanoparticles prepared by CS and plasmid encoding IL-1Ra (pIL-1Ra) have been proven to be effective in transfection of articular chondrocytes and significantly reduce the severity of histologic cartilage lesions [15]. However, low transfection efficiency of CS alone limits the clinical application. Recent researches suggested that transfection efficiency could be improved by combining functional groups with amino groups in chitosan, such as arginine and folic acid [13, 16]. Hyaluronic acid (HA), a natural biodegradable polysaccharide, has aroused general concern. As Lu et al reported, CS/HA/pDNA nanoparticles encoding TGF-β1 can promote chondrocyte adhesion, proliferation, and synthesis of extracellular matrix [17]. Despite the above reports, to our knowledge, there is no study that explores the potential effects of HA-modified CS nanoparticles combined with IL-1Ra gene on synoviocytes.

In the present work, we designed a non-viral gene vector consisting of CS and HA for the targeted delivery of plasmid containing IL-1Ra gene into synoviocytes (CS/HA/pIL-1Ra). We investigated the characterization of CS/HA nanoparticles in different conditions including size, zeta potential, pDNA protection ability, and the release behavior of the loaded pDNA. Furthermore, the experiments in vitro were carried out to examine the subsequent IL-1Ra gene overexpression and its anti-inflammatory effect in inflammation induced by IL-1β in synoviocytes, which have offered insights to explore potential therapeutic strategies for OA synovitis.

## Materials and methods

### Materials

Chitosan (molecular weight, 5 kDa; deacetylation degree, 90%), and chitosanase were purchased from Sigma-Aldrich (USA). Sodium hyaluronate (molecular weight, 35 kDa) was purchased from Freda Biochem Co., Ltd. (Shandong, China). Deoxyribonuclease I (DNase I), SYBR Prime Ex Taq II were obtained from Invitrogen (USA). The streptavidin-biotin-peroxidase complex (SABC) kit and the primary antibodies against matrix metalloproteinase-3 (MMP-3), matrix metalloproteinase-13 (MMP-13), cyclooxygenase-2 (COX-2) and inducible nitric oxide synthase (iNOS) were purchased from Boster (Wuhan, China). Collagenase II, trypsinase, antibiotics, Dulbecco’s modified Eagle’s medium (DMEM)/F12, and fetal bovine serum (FBS) were obtained from Gibco. All reagents used in this study were of analytical grade.

### Preparation of nanoparticles loaded with IL-1Ra encoding pDNA

pDNA expression vector (purchased from Fulengen Co., Ltd., Guangzhou, China) containing a human cytomegalovirus promoter inserted upstream and the sequence of IL-1Ra was propagated in Escherichia coli cells, isolated, and purified. The same plasmid vector consisting of the coding sequence of EGFP and the cytomegalovirus enhancer inserted at the upstream was used as an empty plasmid control. To evaluate the plasmid concentration and purity, the absorption ratio was measured at λ = 260 nm and 280 nm.

The preparation of nanoparticles and the corporation of pDNA were carried out by a simple methodology. Chitosan and sodium hyaluronate were completely dissolved in ultrapure water to produce solutions with good dispersion, respectively. Both the solutions were filtered through a 0.2 μm membrane. Then, put the CS solution into a ultrasound apparatus, meanwhile, the HA solution was added dropwise to the CS solution. The reaction was continued for 15 min to form the complexes of CS/HA via electrostatic interaction. In order to get the optimum CS:HA mass ratio, the complexes were prepared with CS:HA mass ratio at 1:1, 2:1, 3:1, 4:1, 5:1, 6:1, and 7:1. We used a CS concentration of 11.25, 22.5, 33.75, 45, 56.25, 67.5, 78.75 *μ*g/ml, respectively, and a constant HA concentration of 11.25 *μ*g/ml. To fabricate CS/HA/pDNA nanoparticles, 12.5 *μ*g/ml pDNA was added to the CS/HA solution by gentle pipetting and then the mixture was vortexed rapidly for 3-5 s and incubated at 37 °C for 30 min to allow sufficient time for CS/HA/pDNA nanoparticles to form completely.

### Physicochemical characteristics and morphology of nanoparticles

Nanoparticles were prepared in ultrapure water at 25 °C. The average particle size and zeta potential of nanoparticles were measured by a Mastersizer 2000 laser diffractometer (Malvern Instruments, Worcestershire, UK). Each experiment was repeated three times. Prior to Scanning Electron Microscopy (SEM) analysis the CS/HA nanoparticles in solutions were carefully dropped onto a silica surface and were lyophilized overnight, then were sputter coated with a layer of gold. Micrograph was observed using SEM (Hitachi X-650, Japan).

### Gel retardation assay

In order to evaluate the ability of protection by CS/HA on pDNA, CS/HA/pDNA nanoparticles and naked pDNA were incubated with 4 *μ*g/ml DNase I for 30 min at 37 °C. Complexes were prepared at a constant pDNA concentration of 12.5 *μ*g/ml. And CS/HA/pDNA nanoparticles were also exposed to a 2.78 *μ*g/*μ*l chitosanase for 12 h at 37 °C. After incubation, the complex solution was mixed with loading buffer and loaded on a 1% agarose gel containing GelRed in Tris-borate-EDTA buffer. The gel electrophoresis was run at 80 V for 45 min and then photographed using a GDS-8000 (UVP, USA).

### In vitro pDNA release studies

CS/HA/pDNA nanoparticles were incubated in PBS (pH 6.8) at 37 °C in a shaker bath at 100 rpm. At appropriate time intervals (0 d, 3 d, 5 d, 7 d, 9 d, 11 d, 13 d, 15 d), the nanoparticle suspension was centrifuged to collect the supernatant and replaced with equal volumes of fresh PBS solution, and then the supernatant was measured spectrophotometrically at 260 nm (DU640; Beckman, Fullerton, CA, USA).

### Cell culture and transfection

Synovial tissue from Sprague-Dawley rat knee joints (purchased from the Experimental Animal Center of Wuhan University) was isolated and placed into PBS. In brief, the synovial tissue was minced aseptically into small pieces and treated with 0.25% trypsin-containing solution at 37 °C for 1 h. Washed with DMEM and PBS twice, synovial tissue was incubated in 0.2% collagenase with agitation at 37 °C for 4 h. After washing with DMEM, the resulting synoviocytes were suspended in DMEM/F12 medium supplemented with 10% FBS and 1% antibiotics and placed in an incubator at 37 °C and 5% CO_2_. At confluence, cells were trypsinized and split in a 1:3 ratio, then recultured in DMEM/F12 medium. All procedures involving animals in the present study were approved by the Wuhan University Animal Care and Use Committee.

Synoviocytes were seeded into 24-well plates at a density of 1 × 10^5^ cells per well in 500 *μ*l of DMEM/F12. After incubation for 24 h, the medium was removed and the synoviocytes were washed once with PBS. Subsequently, CS/HA/pEGFP and CS/HA/pIL-1Ra nanoparticles solution were added to the cells in DMEM containing 10% FBS and 1% antibiotics. Finally, after incubation for 72 h, the nanoparticles solution was then removed and the protein expression of IL-1Ra in synoviocytes was detected by western blot analysis.

### Cytotoxicity assays

The effect of nanoparticles on the viability of synoviocytes was evaluated using the colorimetric MTS (3-(4,5-dimethylthiazol-2-yl) -5-(3-carboxymethoxyphenyl)-2-(4-sulfophenyl)-2H-tetrazolium) assay. Synoviocytes were seeded, 24 h prior to transfection, in 96-well plates at a density of 1 × 104 cells per well in 100 *μ*l medium. After culture medium was removed, CS/HA/pDNA nanoparticles at concentrations ranging from 0 to 160 *μ*g/ml were immediately added to cells. A group of cells containing only DMEM medium was used as a blank control. After 72 h of co-culture, MTS and phenazine methosulfate were added to synoviocytes followed by incubation for 2 h at 37 °C. Then, the absorbance was measured at 490 nm using a microplate reader.

### Synoviocytes culture and IL-1β treatments

Primary synoviocytes were divided into four groups as blank group, control group, CS/HA/pEGFP group and CS/HA/pIL-1Ra group. The blank group was kept untreated except for the replacement of the medium, and the control group was merely treated with 10 ng/mL IL-1β. Synoviocytes of the CS/HA/pEGFP group and the CS/HA/pIL-1Ra group were treated with CS/HA/pEGFP or CS/HA/pIL-1Ra nanoparticles in the presence of IL-1β (10 ng/ml). After incubation for 72 h, synoviocytes were collected and the results of interest were tested by RT-qPCR and western blotting.

### Real-time quantitative polymerase chain reaction (RT-qPCR)

Total RNA was extracted from synoviocytes by using TRIzol and chloroform reagents according to the manufacturer’s instructions. RNA concentration and purity were determined using spectrophotometer (DU640; Beckman, Fullerton, CA, USA). RNA was transcribed into cDNA using the PrimeScript RT Master Mix kit and amplified by PCR. Genes of interest were quantified, such as MMP-3 (forward: 5’-GGCCATCTCTTCCTTCAG-3’, reverse: 5’-GTCACTTTCTTTGCATTTGG-3’), MMP-13 (forward: 5’-TTCGGCTTAGAGGTGACAGG-3’, reverse: 5’-ACTCTTGCCGGTGTAGGTGT-3’), COX-2 (forward: 5’-CCCTTGGGTGTCAAAGGTAA-3’, reverse: 5’-GCCCTCGCTTATGATCTGTC-3’), iNOS (forward: 5’-CTGCTTGAGGTGGGCGG-3’, reverse: 5’-GTGACTCTGACTCGGGACGCC-3’). The GAPDH was used as a normalization control (forward: 5’-TGTCGTGGAGTCTACTGGTG-3’, reverse: 5’-GCATTGCTGACA ATCTTGAG-3’). The RT-qPCR assay was performed using iCycler iQ RT-qPCR detection system (BioRad) with the SYBR Prime Ex Taq II kit. 20 *μ*L reaction volume containing 2 *μ*L of cDNA, 0.8 *μ*L of forward and reverse primer each, 10 *μ*L of SYBR Prime Ex Taq II, and 6.4 *μ*L of nuclease-free water. Reactions were performed using the following conditions: initial denaturation for one cycle at 95 °C for 30 s; 40 cycles of denaturation at 95 °C for 5 s, annealing and extension at 60 °C for 34 s. Specific PCR products were confirmed by melting curve analysis.

### Western blot analysis

Proteins were extracted from synoviocytes and protein concentrations were adjusted using the bicinchoninic acid protein assay kit (Fermentas, Thermo Fisher Scientific, Waltham, MA, USA). Every well in the 10% separating SDS-PAGE was loaded with 20 *μ*L protein. SDS-PAGE was used to separate equal quantities of protein and separated proteins were transferred to nitrocellulose membranes (Fermentas, Thermo Fisher Scientific, Waltham, MA USA). The membranes were treated with PBS containing 5% nonfat dry milk, and incubated with primary antibodies [MMP-3 (1:1,000, Boster, Wuhan, China), MMP-13 (1:1,000, Boster, Wuhan, China), COX-2 (1:1,000, Boster, Wuhan, China) and iNOS (1:1,000, Boster, Wuhan, China)] overnight at 4 °C. After washing, the membranes were treated with horseradish peroxidase-conjugated secondary antibodies, followed by visualization using an enhanced chemiluminescence kit (Fermentas, Thermo Fisher Scientific, Waltham, MA, USA). Blots were scanned using a gel imaging system (GelDoc-It 310, UVP Co., Upland, CA, USA) and densitometric analyses were performed using Image Lab 4.1 software (Bio-Rad Laboratories, Hercules, CA, USA).

### Statistical analysis

All data are expressed as the mean ± standard deviation, and statistical analyses were performed using the statistical software package SPSS 20.0. Comparisons among groups were analyzed using one-way analysis of variance (ANOVA), followed by Student–Newman–Keuls test. *p* < 0.05 was considered to be significant.

## Results

### Physicochemical characteristics of CS/HA nanoparticles

**Fig. 1:**
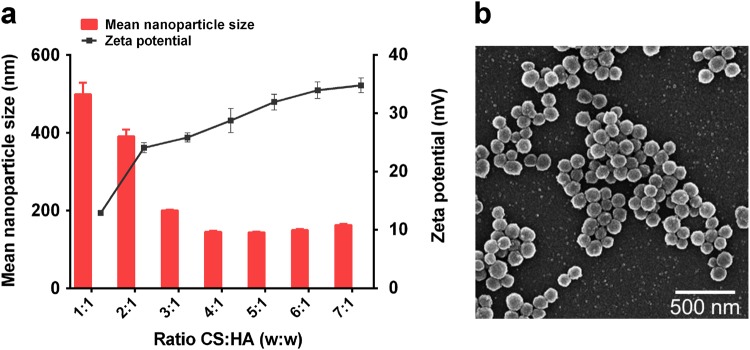
Characteristics and morphology of nanoparticles. **a** Nanoparticle size and zeta potential of CS/HA complexes at various mass ratios ranging from 1:1 to 7:1 (CS:HA, w/w). **b** A representative image of CS/HA nanoparticles taken by scanning electron microscopy (SEM), which shown here was a lyophilized nanoparticle sample with a mass ratio of CS:HA of 4:1 (scale bar = 500 nm)

The relationship between the mass ratio of CS to HA and the resulting mean nanoparticle size and zeta potential were shown in Fig. 1a. An increase in CS amount resulted in the formation of significantly smaller CS/HA nanoparticles, and the smallest particle size (144.9 ± 2.8 nm) was formed with a 4:1 ratio of CS:HA. As proved by the statistical differences in Fig. 1a, thae zeta potential became more positive with the amount of CS increased. The highest zeta potentials were obtained at CS:HA mass ratios higher than 2:1, reaching around + 28 mV with a 4:1 ratio of CS:HA. The morphologies of CS/HA nanoparticles taken by SEM were shown in Fig. 1b. The representative images showed that these particles had a good dispersion and a regular spherical shape with a diameter of 100-300 nm.

### Electrophoresis assay

**Fig. 2:**
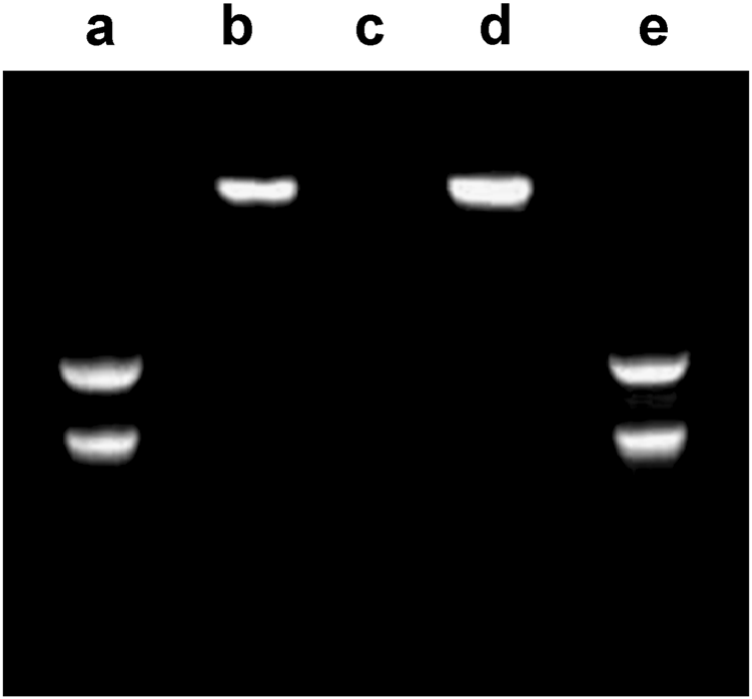
Gel electrophoresis of CS/HA/pIL-1Ra nanoparticles. Lane a: normal naked pIL-1Ra; Lane b: CS/HA/pIL-1Ra nanoparticles; Lane c: naked pIL-1Ra digested by DNase I; Lane d: CS/HA/pIL-1Ra nanoparticles digested by DNase I; Lane e: CS/HA/pIL-1Ra nanoparticles digested by chitosanase

As shown in Fig. 2, lane b demonstrated that the pDNA migration was totally retained which indicated the complete combination of pDNA with CS/HA via an electrostatic interaction. The fluorescent light from the lane containing naked pDNA disappeared completely after adding DNase I, whereas the fluorescence intensity decreased only slightly from the lane containing CS/HA/pIL-1Ra nanoparticles. However, following digestion with chitosanase, the pDNA could be released from the nanoparticles and could be viewed in lane e.

### The pDNA release curve of CS/HA/pDNA

**Fig. 3:**
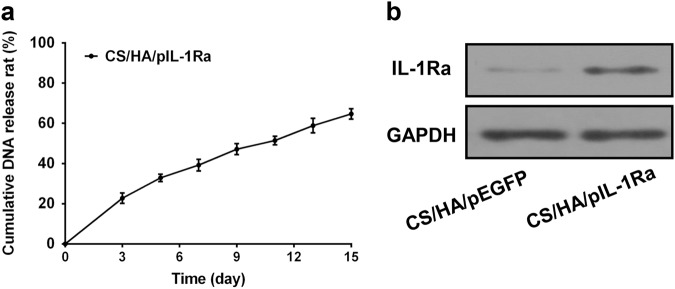
Released kinetics character of CS/HA/pIL-1Ra nanoparticles and the expression of IL-1Ra in synoviocytes. **a** Cumulative pIL-1Ra release curve of CS/HA/pIL-1Ra nanoparticles as a function of time up to 15 days. Values were expressed as the mean ± standard deviation. **b** The protein expression of IL-1Ra in synoviocytes was detected by western blot analysis after coculturing with CS/HA/pEGFP or CS/HA/pIL-1Ra nanoparticles for 72 h. GAPDH was used as an internal control

To further investigate the released kinetics character of CS/HA/pIL-1Ra nanoparticles, Fig. 3a depicted the pDNA release curve which exhibited a small burst of about 22% in the first three days and followed by a constant release rate. The pDNA release remained steady with a slow increase, reaching approximately 65% after 15 days. These data suggested that CS/HA nanoplexes exhibited considerable buffering capacity and contributed to the sustaining endocytosis by cells.

### IL-1Ra expression

We used western blot analysis to confirm the overexpression of IL-1Ra after 72 h post-transfection. As demonstrated in Fig. 3b, a rise in protein expression of IL-1Ra was observed in group CS/HA/pIL-1Ra nanoparticles, while the CS/HA/pEGFP group produced a very low IL-1Ra level. The expression of GAPDH was used as a protein loading control.

### Cell viability

**Fig. 4:**
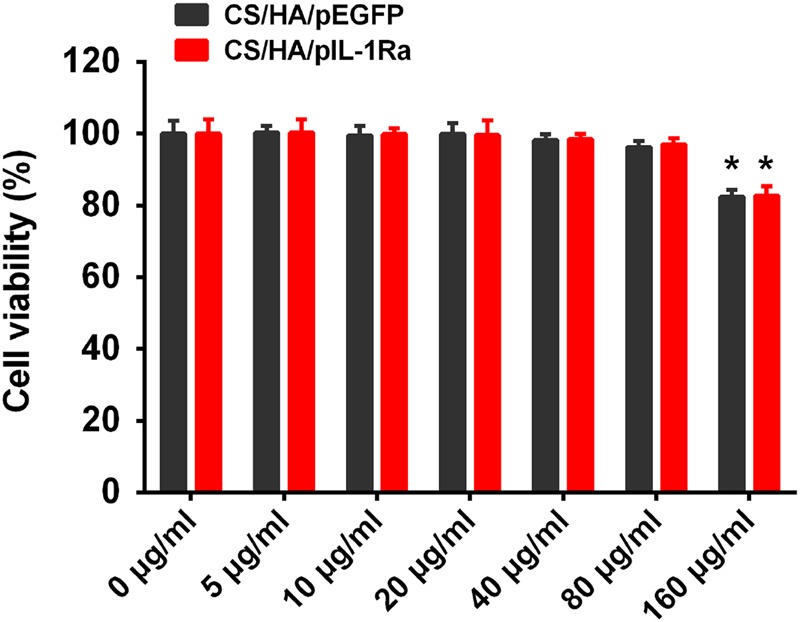
Cell viability of CS/HA/pDNA nanoparticles. Cell viability of synoviocytes after incubation with CS/HA/pEGFP and CS/HA/pIL-1Ra nanoparticles at different concentration from 0 to 160 *μ*g/ml after 72 h. *p < 0.05, compared with 0 *μ*g/ml

We evaluated the cytotoxicity of CS/HA/pEGFP and CS/HA/pIL-1Ra nanoparticles in synoviocytes by measuring their metabolic activity. The cell viabilities in the concentration range of 0-160 *μ*g/ml after 72 h exposure were shown in Fig. 4. CS/HA/pIL-1Ra nanoparticles showed similar low cytotoxicity with CS/HA/pEGFP nanoparticles. Both CS/HA/pEGFP and CS/HA/pIL-1Ra nanoparticles displayed no difference in cytotoxicity with the complex dose increased to 80 *μ*g/ml, but there was increased cytotoxicity when cells were cultured in 160 *μ*g/ml nanoparticles (*p < 0.05). Thus, the concentration of 80 *μ*g/ml nanoparticles was selected for the next experiments.

### CS/HA/pIL-1Ra nanoparticles reduced MMP-3, MMP-13, COX-2 and iNOS expressions

**Fig. 5:**
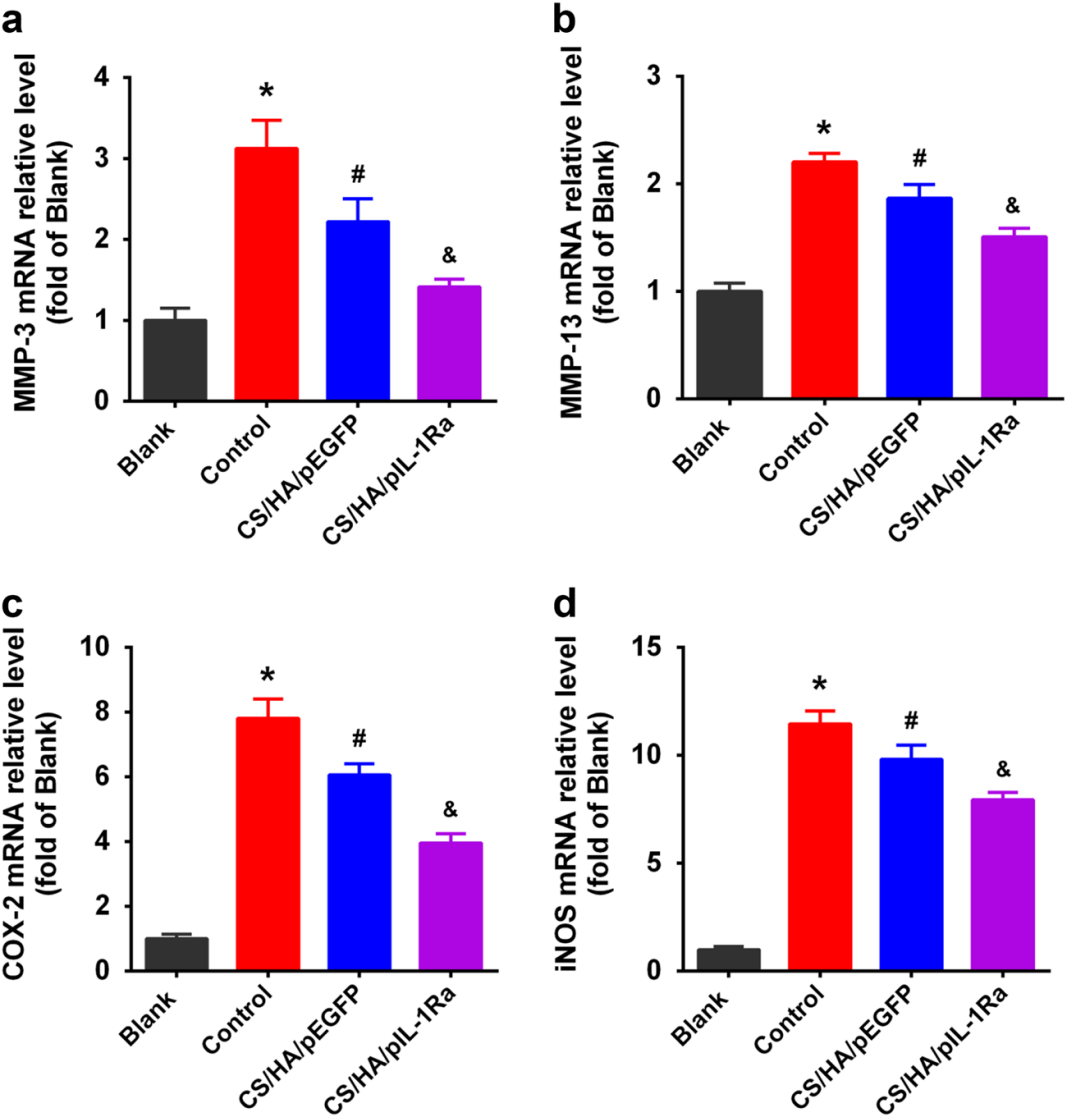
Effect of CS/HA/pDNA nanoparticles on IL-1β-induced mRNA expressions of MMP-3, MMP-13, COX-2 and iNOS. When synoviocytes were treated with CS/HA/pDNA nanoparticles in the presence of IL-1β, IL-1β-induced mRNA expressions of MMP-3 **a**, MMP-13 **b**, COX-2 **c** and iNOS **d** within 72 h were analyzed by RT-qPCR. Bars represent the means ± SEM (n = 3). *p < 0.05 versus the blank group; ^#^*p* < 0.05 versus the control group; ^&^*p* < 0.05 versus the CS/HA/pEGFP group

**Fig. 6:**
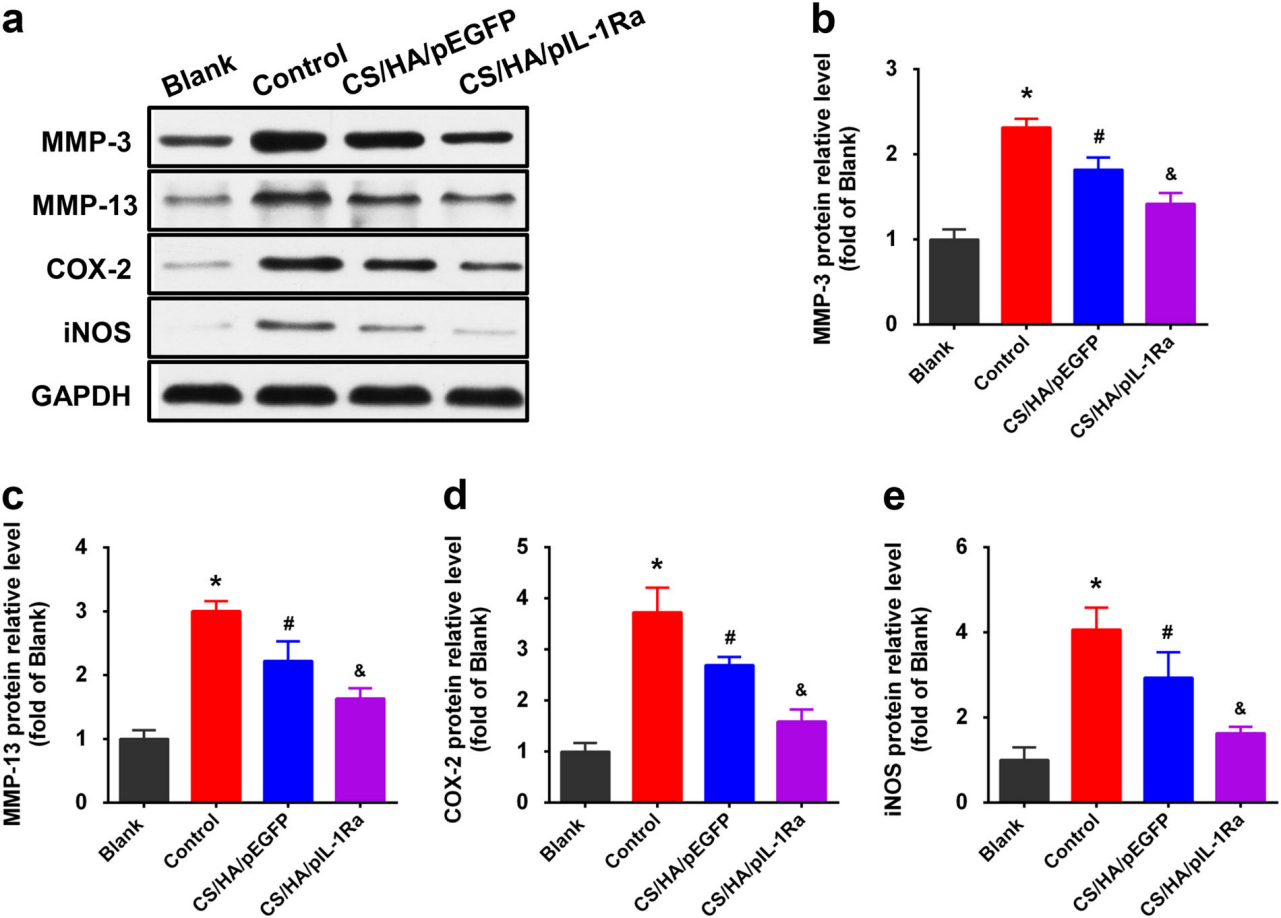
Effect of CS/HA/pDNA nanoparticles on IL-1β-induced protein expressions of MMP-3, MMP-13, COX-2 and iNOS. **a** In synoviocytes treated with CS/HA/pDNA nanoparticles and simultaneous IL-1β stimulation, the levels of MMP-3, MMP-13, COX-2 and iNOS proteins were detected by western blot analysis. The quantifications of differential expressions of MMP-3 **b**, MMP-13 **c**, COX-2 **d** and iNOS **e** were described. Bars represent the means ± SEM. *p < 0.05 versus the blank group; ^#^*p* < 0.05 versus the control group; ^&^*p* < 0.05 versus the CS/HA/pEGFP group

In the end, we investigated the treatment for anti-inflammatory effect in inflammation induced by IL-1β in synoviocytes. In the control group, we incubated the cells with the indicated concentrations of IL-1β (10 ng/ml) for 72 h. As shown in Fig. 5a, b, RT-qPCR showed that MMP-3, MMP-13 mRNA expressions were strongly enhanced compared with those in the blank group (*p < 0.05). Since IL-1β is known to induce the expression of COX-2 and iNOS, which leads to the production of PGE_2_ and NO [18], we also observed the stimulation of COX-2 and iNOS mRNA expressions in these synoviocytes (*p < 0.05; Fig. 5c, d). In the CS/HA/pDNA group, synoviocytes were treated with CS/HA/pDNA nanoparticles in the presence of IL-1β (10 ng/ml). CS/HA/pEGFP nanoparticles decreased the the expressions of MMP-3, MMP-13, COX-2 and iNOS mRNA (^#^*p* < 0.05), but not as strong as the CS/HA/pIL-1Ra group (^&^*p* < 0.05). Figure 6 showed that the levels of MMP-3, MMP-13, COX-2 and iNOS proteins were detected by western blot analysis in various experimental groups. Consistent with the results of RT-qPCR, the protein expressions of above-mentioned were also attenuated in the CS/HA/pEGFP group (^#^*p* < 0.05) and markedly attenuated in the CS/HA/pIL-1Ra group (^&^*p* < 0.05).

## Discussion

Although OA has been characterized by main alterations in the composition, structure and function of the articular cartilage, the concept of synovial inflammation contributing to the entire stage of OA has recently been emphasized. Specifically, histological changes of synovium include a range of pathologic changes, such as synovial lining hyperplasia, infiltration of inflammatory cell, increased vascularity and fibrin deposition that are associated with clinical symptoms and also reflect the degree of cartilage degradation [19, 20]. Hence, synovium-targeted gene therapy may help to sustainable alleviate the symptoms of the disease and reduce structural progression. In our study, we showed that CS/HA nanoparticles fulfilled the main criteria required for gene delivery, such as an appropriate particle size, positive zeta potential and DNA encapsulation ability, and also exhibited considerable buffering capacity. We also demonstrated that CS/HA/pDNA nanoparticles encoding IL-1Ra were able to increase IL-1Ra expression in primary synoviocytes, and reduce the mRNA and protein levels of MMP-3, MMP-13, COX-2 and iNOS in IL-1β-induced synoviocytes.

Although gene delivery systems can be viral or non-viral, non-viral vectors are advantageous in some respects such as their safety, low immunogenicity, and convenient application [21]. CS is a biocompatible polysaccharide which is similar to glycosaminoglycan that existed in human cartilage [22]. It has been extensively used for cell transfection and tissue engineering [23, 24]. In addition, since high adhesive properties have been observed with chitosan [25], intra-articular injection of such vectors may contribute to targeting synovial cells. HA is an anionic biopolymer present in the extracellular matrix and synovial fluids [26]. HA can also specifically recognize CD44 receptor whose expression is strongly increased in the synoviocytes and chondrocytes from the patients with OA [27]. The specific binding properties of HA for the CD44 receptor offer an access to the target cells through receptor-mediated endocytosis pathway [28]. Because CS and HA are oppositely charged, the complex can be formed via electrostatic interaction. In addition, both of them are expected to interact through hydrogen bonds and other intermolecular forces [29]. Therefore, CS/HA complex is appealing as a vector for specifically targeting synoviocytes and allowing endocytosis of nanoparticles with much fewer side effects compared to non-targeted treatments [30].

In the present study, the influence of the mass ratio of CS and HA on the physicochemical characteristics of the resulting nanoparticles were evaluated. Two properties are necessary to assure nanoparticles uptake by cells: size and zeta potential [16]. Our results indicated that the size and zeta potential of CS/HA nanoparticles depended on their composition, with a decrease in size and an increase in zeta potential with increasing CS content. According to Fig. 1a, we showed that the 4:1 ratio was an optimal choice of CS:HA. It was in accordance with a previous investigation, which proved that the combination of HA with ultra low molecular weight chitosan presents high transfection efficiency [31]. In the electrophoresis assay, CS/HA nanoparticles could encapsulate pDNA completely, and using DNase as a model enzyme, the result showed pDNA was not released from complexes, indicating that CS/HA nanoparticles could protect pDNA efficiently from degrading by nuclease which is one of the crucial factors for efficient gene delivery [32]. Moreover, the pDNA was released from CS/HA complex and could be seen after addition of chitosanase, suggesting that synthetic process did not affect the integrity of encapsulated pDNA. We also showed that CS/HA/pIL-1Ra complex was a well-controlled system capable of releasing pDNA for more than 15 days that supported the long-term gene therapy at a localized site, consistent with a previous report that CS/HA/pTGF-β1 nanparticles were able to release pTGF-β1 for a lengthy period [17]. Then, the successful overexpression of IL-1Ra in synoviocytes at 72 h post-transfection was proved by western blot detection. This result supported the earlier study which effectively transfected luciferase pDNA into macrophages by CS/HA complex [33]. Therefore, the above results suggested that the CS/HA/pIL-1Ra nanoparticles we synthesized were suitable for gene delivery and able to successfully transfect IL-1Ra gene into synoviocytes.

IL-1β is mainly produced by activated synoviocytes, mononuclear cells and chondrocytes [34]. After binding to IL-1R in synoviocytes, IL-1β activates numerous transcriptional factors and regulates inflammatory response through NF-κB and MAPKs signaling pathway, inducing the production of MMPs, PGE_2_, NO and other catabolic factors [35, 36]. MMPs secreted by OA synovium are known to be important factors in breaking down collagen and proteoglycans [37]. PGE_2_ and NO synthesized via the upregulation of COX-2 and iNOS gene expressions have been suggested to lead to cartilage degradation, inhibition of matrix synthesis, and chondrocyte apoptosis [38, 39]. Considering our results, it has been demonstrated that the protein and mRNA expressions of MMP-3, MMP-13, COX-2 and iNOS in IL-1β stimulated synoviocytes were indeed inhibited by CS/HA/pEGFP nanoparticles, which was consistent with previous reports that chitosan alone exhibited anti-inflammatory effects in vitro and in vivo [40, 41]. Furthermore, we found that the combination of IL-1Ra gene and CS/HA nanoparticles was effective, resulting in more significant anti-inflammatory effects compared with those of CS/HA/pEGFP nanoparticles, suggesting that the overexpression of IL-Ra by CS/HA/pIL-1Ra nanoparticles could substantially block the inflammatory effects of IL-1β.

The purpose of this program of research was to seek insights into what effects CS/HA/pIL-1Ra nanoparticles have on synovitis induced by IL-1β in vitro, In our future research, we will observe the changes in chondrocytes phenotype when co-cultured with OA synoviocytes that pre-administrated with CS/HA/pIL-1Ra nanoparticles. Meanwhile, we will also explore the effects of CS/HA/pIL-1Ra nanoparticles on synoviocytes and chondrocytes in OA animal models.

## Conclusion

The current study prepared the CS/HA/pIL-1Ra nanoparticles through electrostatic interaction. Particle size and zeta potential measurement, SEM, gel retardation assay and pDNA release kinetics were applied to reveal that the CS/HA/pIL-1Ra nanoparticles had appropriate physicochemical characteristics for gene delivery. Furthermore, our cytotoxicity results indicated that the CS/HA/pIL-1Ra nanoparticles displayed no cytotoxic effect on synoviocytes below the concentration of 80 *μ*g/ml nanoparticles. The in vitro transfection studies were carried out to certify that CS/HA complex as a gene vector was feasible in primary synoviocytes, and CS/HA nanoparticles transferring the IL-1Ra gene were functional in synoviocytes, which could effectively reduce the inflammatory effects stimulated by IL-1β. Our results suggested that the CS/HA/pIL-1Ra nanoparticles might have a great potential for the development of therapeutic intervention in inflammation induced by IL-1β in synoviocytes.
